# Disrupted gut microbiota promotes the progression of chronic kidney disease in 5/6 nephrectomy mice by *Bacillus pumilus* gavage

**DOI:** 10.3389/fcimb.2025.1548767

**Published:** 2025-03-18

**Authors:** Fei Chen, Hailin Zhang, Qianqian Wei, Jie Tang, Lixia Yin, Yanan Ban, Qifan Zhou

**Affiliations:** ^1^ Blood Purification Centre, The Affiliated Lianyungang Hospital of Xuzhou Medical University, Lianyungang, China; ^2^ Blood Purification Centre, Lianyungang Clinical College of Nanjing Medical University, Lianyungang, China; ^3^ Nursing Department, Yixing Traditional Chinese Medicine Hospital, Wuxi, China; ^4^ Blood Purification Centre, The First People’s Hospital of Lianyungang, Lianyungang, China

**Keywords:** *B. pumilus*, chronic kidney disease, dysbiosis, gut-kidney axis, gut metabolism, gut microbiota, uremic toxins

## Abstract

**Background:**

Our previous study identified differences in the gut microbiota between patients with chronic kidney disease (CKD) and healthy individuals. We observed that antibiotic-treated mice exhibited symptoms similar to those of patients with CKD after receiving a gut microbiota transplant from patients with CKD. *Bacillus pumilus* (*B. pumilus*), an alien microorganism to both human and mouse gut microbiota, possesses antibiotic properties that can alter the microbial community structure. Therefore, this study aimed to explore how changes in the gut microbiota structure induced by the oral gavage of B. pumilus affect the progression of CKD. We sought to identify the gut microbes and metabolic pathways associated with CKD to lay the groundwork for future clinical probiotic applications in patients with CKD.

**Methods:**

We constructed sham-operated and 5/6 nephrectomy mice as the sham control (SC) and CKD models, respectively. CKD models were divided into a control group (CG) and an intervention group (IG). After 16 weeks of normal feeding, the IG were treated with B. pumilus by oral gavage, while SC and CG were treated with PBS once daily, 5 days per week, for 7 weeks. Fecal samples were collected for 16s rRNA sequencing and metabolomic analysis, kidneys were harvested for histological examination, and the colon was used for RT-PCR analysis.

**Results:**

*B. pumilus* intervention exacerbated gut microbial homeostasis in CKD mice and increased serum creatinine and urea nitrogen levels, further aggravating kidney damage. 16s rRNA and metabolomic analysis revealed that *Parvibacter* and *Enterorhabdus* were probiotics related to kidney function, while *Odoribacter* was associated with kidney injury. Metabolomic analysis showed that glycerophospholipid and lysine metabolism were upregulated in CKD model mice, correlating with kidney damage.

**Conclusion:**

This study shows that changes in the gut microbiota can affect the kidneys through gut metabolism, confirming that the lack of probiotics and the proliferation of harmful bacteria leading to gut microbiota dysbiosis are drivers of CKD progression. Our findings provide a basis for clinical interventions using gut microbes and offer a reference for targeted probiotic therapy.

## 1 Introduction

Chronic kidney disease (CKD) is a progressive and debilitating condition resulting from various factors that lead to irreversible changes in kidney structure and function. Most patients with end-stage CKD rely on hemodialysis or kidney transplantation (Brody, 2023). Since the concept of the gut-kidney axis was established, numerous studies have explored the impact of gut microbiota on kidney diseases, providing insights and evidence for the connection between the gut and kidneys (Ai et al., 2023). Our preliminary findings revealed alterations in the gut microbiota of patients with end-stage kidney disease undergoing hemodialysis (Zhou et al., 2022). Recipient mice that received fecal suspensions from these patients exhibited disease characteristics similar to those of the donors (Tang et al., 2023). Our findings confirmed the correlation between gut microbiota and CKD, suggesting that changes in the gut microbiota could potentially influence disease progression.

Although we have established that transplanting disrupted gut microbiota from CKD patients can induce CKD-like symptoms, it remains unclear whether alterations in the original gut microbiota homeostasis can affect the progression of CKD symptoms. Therefore, to simulate the change in gut microbiota homeostasis, we constructed a CKD mouse model and used oral gavage with *Bacillus pumilus* (*B. pumilus*) to alter the native gut microbiota homeostasis of these models, observe disease progression, and identify the mechanisms by which changes in the gut microbiota drive disease progression. *B. pumilus*, a widely prevalent gram-positive bacillus in nature, is not part of the gut microbiota (Kotowicz et al., 2019) and is known to secrete metabolites that inhibit bacterial growth (Chu et al., 2019). Antibiotics, which are selectively antimicrobial and absorbable by the gut, lack the biological activity to adequately mimic natural changes in the gut microbiota. *B. pumilus*, with its inherent antimicrobial activity and inability to colonize the gut, serves as an ideal catalyst for altering the gut microbiota. Therefore, this study aimed to alter the gut microbiota homeostasis of CKD model mice using *B. pumilus*, observe the impact of these changes on disease progression in CKD mice, and screen for gut microbes associated with CKD.

## 2 Materials and methods

### 2.1 Animals and experiment design

Male C57BL/6 mice, aged 8 weeks (n=21), were procured from Jiangsu Huachuang Sino Medical Technology Co., Ltd. and maintained in a specific pathogen-free (SPF) environment with controlled temperature (24 ± 1℃) and humidity (60 ± 5%). After a one-week acclimatization period, the mice were subjected to anesthesia using 2% isoflurane for the ligation of the upper and lower one-third of the left kidney, employing 3-0 sutures. One week subsequent to the ligation, the right kidney was completely excised via the same procedure. Post-surgical, the mice were monitored for an additional 16 weeks. Orbital blood samples were collected under anesthesia to measure blood creatinine and urea nitrogen, thereby assessing the model’s efficacy (Nishiyama et al., 2019). Within the first week post-surgery, four 5/6 nephrectomy (Nx) mice succumbed. Once the model was validated, the sham-operated group (SG, n=7) served as the control. The surgery group was randomly assigned to either a control group (CG, n=5) or an intervention group (IG, n=5). The SG and CG received daily gavage treatments with 0.4 ml of PBS solution, whereas the IG received 0.4 ml of B. pumilus bacterial solution, administered five times per week for seven weeks. We collected fecal and blood samples in the end of intervention, and then cut the remaining kidney and the bottom of colon tissue for the further analysis. All animal experimental protocols were approved by the Institutional Animal Care and Use Committee of Kangda College of Nanjing Medical University (Application Number: 23004, Approval Number: IACUC-23XS003).

### 2.2 Bacterial culture and collection

*B. pumilus* was provided by the Microbiology Laboratory of the First People’s Hospital of Lianyungang. The collected strains were stored in bacterial preservation tubes at -80°C. Prior to each subculture, the strain was confirmed as *B. pumilus* using mass spectrometry. Before use, the strains were revived from the preservation tubes by inoculation onto blood agar plates and incubated at 37°C for 48 h. Subsequently, sterile PBS was used to prepare a *B. pumilus* bacterial suspension, and the bacterial concentration was determined to be 2.1 McFarland units, which corresponded to approximately 6 × 10^8^ colony-forming units (cfu) per mL. The suspension was prepared on the same day and transported for oral gavage to the mice.

### 2.3 Histological staining analysis

Upon completing the experiment, the kidneys were immediately isolated from euthanized mice, fixed in 10% formalin, and dehydrated using ethanol and xylene. Kidney tissues were then embedded in paraffin and sectioned into thin slices. The sections were treated to remove the paraffin, stained with hematoxylin and eosin (HE), dehydrated, cleared, mounted, and imaged under a light microscope. To assess renal abnormalities, periodic acid-Schiff (PAS) and Masson’s trichrome staining were used to observe glomerular sclerosis and fibrosis, respectively (Wang et al., 2020). The severity of the lesions was scored from 0 to 4 based on the degree of abnormality: “0.5 points” for minimal or trace amounts, “1 point” for mild or small amounts, “2 points” for moderate or moderate amounts, “3 points” for severe or large amounts, “4 points” for very severe or extensive amounts, and “0 points” for normal tissue.

### 2.4 Rt-PCR

We followed the guidelines specified by the producer to isolate RNA from the colonic tissue utilizing the TRIzol reagent provided by Invitrogen. And then we use a reverse transcription kit, from Vazyme, China, to generate complementary DNA (cDNA) from the isolated RNA. Quantitative PCR was performed using an ABI 7300 Real-Time PCR System (Applied Biosystems, USA) to measure the expression of the colon tight junction protein *zonula occludens-1* (*ZO-1*) in colon tissue. The gene sequences used for *ZO-1* were forward: GCC GCT AAG AGC ACA GCA A and reverse: GCC CTC CTT TTA ACA CAT CAGA.

### 2.5 Fecal microbiota analysis

#### 2.5.1 DNA extraction and PCR amplification

We employed the E.Z.N.A.^®^ soil DNA Kit produced by Omega Bio-tek, Norcross, GA, U.S. to isolate genomic DNA from stool samples, following the supplier’s guidelines. Then we use the NanoDrop2000 spectrophotometer (Thermo Scientific, United States) to assess the DNA’s purity and quantity via 1.0% agarose gel electrophoresis. The DNA samples were preserved at -80℃ for future analysis. The variable V3-V4 segments of the bacterial 16S rRNA gene were targeted for amplification through using the primers 338F (5’- ACT CCT ACG GGA GGC AGC AG-3’) and 806R (5’- GGA CTA CHV GGG TWT CTA AT-3’) (Liu et al., 2016) on a T100 PCR thermal cycler from BIO-RAD in the USA. The cleansed amplicons were mixed in equal molar concentrations and subjected to paired-end sequencing by the Illumina Nextseq2000 platform.

#### 2.5.2 Date processing

The original FASTQ files were de-multiplexed by an internal Perl script, then we used fastp version 0.19.6 (Chen et al., 2018) to filter its quality and FLASH version 1.2.7 (Magoč and Salzberg, 2011) to merge them. The criteria were as follows: (i) Within a 50 bp sliding window, reads were truncated if the average quality score of any site was less than 20, and reads were discarded if their length was less than 50 bp or the reads contained ambiguous characters; (ii) We assembled overlapping sequences longer than 10 bp according to their overlapping sequences, with a maximum mismatch rate of 0.2; if the reads could not be assembled, it would be discarded; (iii) We differentiated samples by barcode and primers and adjusted sequence orientation to achieve exact barcode matching, allowing only two nucleotide mismatches. We clustered the optimized sequences into operational taxonomic units (OTUs) using UPARSE7.1 (Edgar, 2013), and the sequence similarity of each OTU was 97%. The most abundant sequence of each OTU was taken as the representative sequence. To minimize the impact of sequencing depth on alpha and beta diversity measures, the number of 16S rRNA gene sequences was reduced to 48,117 for each sample.

#### 2.5.3 Date analysis

We calculated sparse curves and alpha diversity indices by analyzing OTU information using Mothur v1.30.1, including PD, Shannon, ACE, Simpson, and Chao (Schloss et al., 2009). The similarities between microbial communities in different samples were determined by principal coordinate analysis (PCoA) based on Jaccard dissimilarity using the Veganv2.5-3 package. The PERMANOVA test was used to assess the percentage of variation explained by the treatment, along with its statistical significance, using the Vegan v2.5-3 package. We used the linear discriminant analysis (LDA) (Segata et al., 2011) (http://huttenhower.sph.harvard.edu/LEfSe) effect size (LEfSe) to identify significantly enriched bacterial taxa (phylum to genus) between groups (LDA score >3.5, P < 0.05). We filtered for differential bacteria under the condition that species must have at least five sequences in at least three samples. Owing to multicollinearity among the 15 mouse parameters, the variance inflation factor (VIF) of each variable was estimated using the vif function in the car package (https://cran.r-project.org/web/packages/car/car.pdf). We explore the internal community relationships between samples by constructing a co-occurrence network (Barberán et al., 2012). A correlation between two nodes was considered statistically significant if the Spearman’s correlation coefficient was > 0.6 or < -0.6 and the *p*-value was less than 0.05.

### 2.6 Metabolomic profiling

#### 2.6.1 Sample processing

A 50 mg stool sample was added into a 2 mL centrifuge tube equipped with a 6 mm grinding ball. For metabolite extraction, 400 μL of the extraction mixture (methanol:water = 4:1, v:v) with an inclusion of 0.02 mg/mL internal standard (L-2-chlorophenylalanine) was introduced. The samples underwent grinding with the Wonbio-96c (Shanghai Wonbio Biotechnology Co., Ltd) cryogenic tissue mill for 6 minutes at -10℃ and 50 Hz, succeeded by cold ultrasonication for 30 minutes at 5℃ and 40 kHz. Subsequently, the samples were kept at -20℃ for 30 minutes and then centrifuged for 15 minutes at 4℃ with a force of 13,000 g. The supernatant liquid was pipetted into a vial for subsequent LC-MS/MS examination. The LC-MS/MS examination was performed on a Thermo UHPLC-Q Exactive HF-X platform, fitted with an ACQUITY HSS T3 column (100 mm x 2.1 mm in diameter, 1.8 μm; Waters, USA).

#### 2.6.2 Data analysis

Raw liquid chromatography-mass spectrometry (LC-MS) data were preprocessed using Progenesis Ql software by Waters Corporation, Milford, USA, to generate a CSV-formatted three-dimensional data array. This array detailed sample information, metabolite names, and their corresponding mass spectrometry response intensities. Data preprocessing involved the removal of peaks from internal standards and deleted false-positive peaks, including noise, column bleed, and peaks from derivatized reagents. The resulting data array was then refined and pooled for peak analysis. Metabolite identification was achieved through database queries in HMDB (http://www.hmdb.ca/), Metlin (https://metlin.scripps.edu/), and Majorbio. Before performing statistical analysis on each group of metabolite data, we removed those metabolite data whose zero values occupy more than 20% in any group. For missing values, we filled them with the minimum value from the original matrix to minimize possible errors.

Mass spectrometry peak intensities were normalized by the total sum normalization technique to correct for potential errors due to sample handling and instrument variability, resulting in a standardized data array. We excluded the variables from the OC samples with a relative standard deviation exceeding 30%. The data were then log10 transformed to obtain the final data array for subsequent analysis. The R package “ropls” (version 1.6.2) was utilized for principal component analysis (PCA) and orthogonal partial least squares discriminant analysis (OPLS-DA), which included a seven-cycle iterative validation to assess model stability. The filtration condition of significant metabolites was that variable importance in projection (VIP) score above 1.5 and p-value less than 0.05, the p-value from the Student’s t-test. Differential metabolites between groups were correlated with their biochemical pathways through metabolic enrichment and pathway analysis, using the Kyoto Encyclopedia of Genes and Genomes (KEGG) database (http://www.genome.jp/kegg/). These metabolites were categorized based on their associated pathways and functions, providing insights into the metabolic differences between the groups.

### 2.7 Statistical analysis

Data normally distributed were analyzed statistically via one-way ANOVA and independent samples t-tests. For non-parametric comparisons among two independent samples, the Mann-Whitney U test was applied, whereas the Kruskal-Wallis H test was utilized for comparisons involving multiple independent samples. The correlation between variables was assessed using Spearman’s rank correlation analysis. To control for multiple comparisons, in the analysis of alpha diversity, particularly in the screening and comparison of differential microorganisms and metabolites, we employed FDR (False Discovery Rate) correction to reduce the false positive rate in our multiple statistical analysis. Analysis was conducted using SPSS (IBM SPSS Statistics v27.0), and data visualization was performed with GraphPad Prism v8.0.

## 3 Result

### 3.1 *B. pumilus* disrupts the gut microbiota structure in 5/6 Nx mice

**Figure 1:**
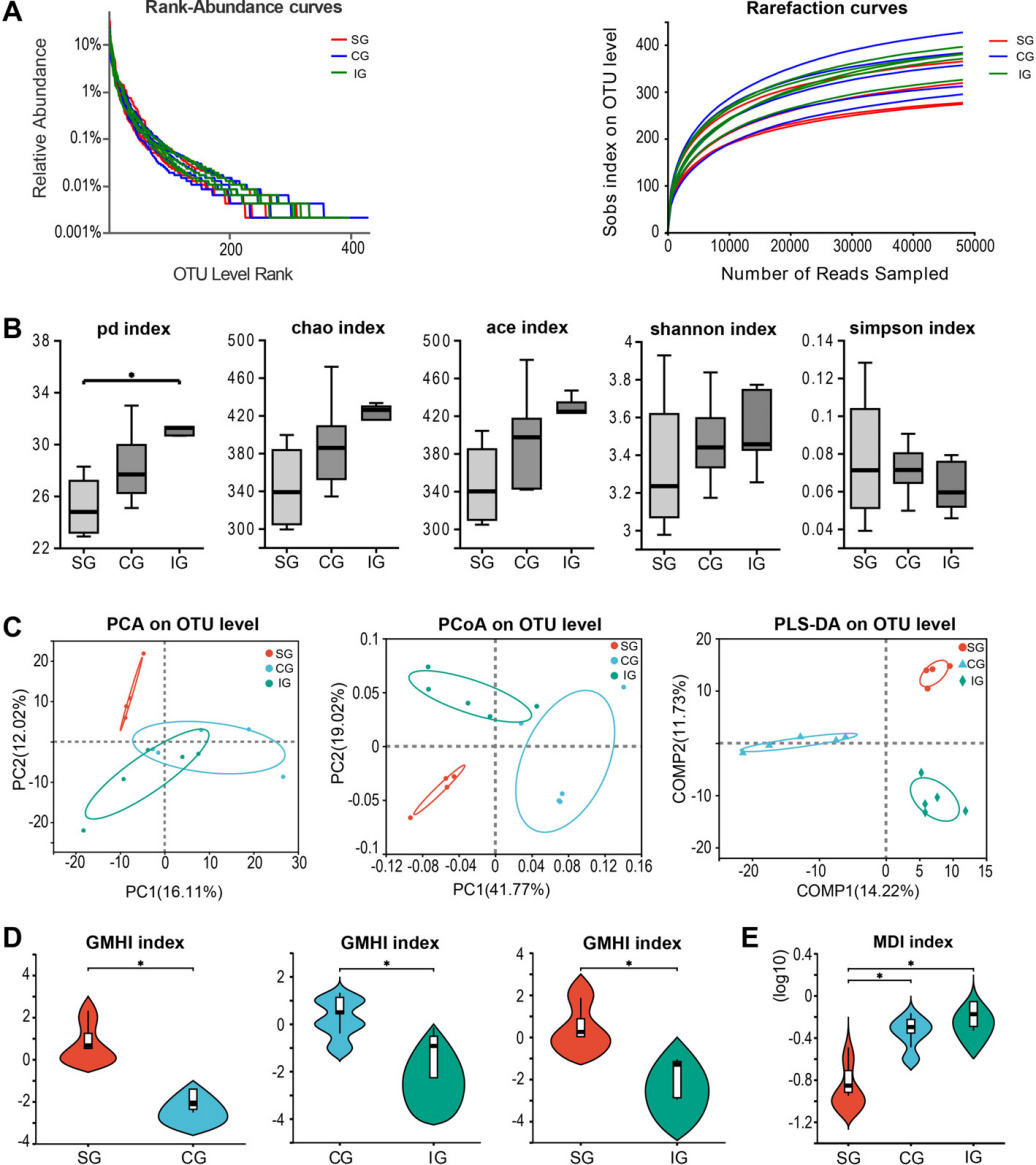
Changes in intestinal flora among SG, CG, and IG after Bacillus pumilus intervention. **(A)** Rank abundance curve and dilution curve showing microbial diversity in the three groups of samples; **(B)** Comparison of α diversity analysis of the three groups of microbial samples; **(C)** Diversity analysis among the three groups of samples; **(D)** Gut microbiome health index (GMHI) between the two groups; **(E)** Dysbiosis index among the three groups. * indicates *p*<0.05.

First, we conducted quality assessments of all the detected microbial samples. Rarefaction and dilution curve analyses based on the Sobs index showed a gradual plateau, indicating that the sequencing sample size was sufficient for data analysis (**Figure 1A**). After excluding a deflected sample whose kidney was damaged during the molding operation in the SC, we further observed the similarity among samples through hierarchical clustering analysis, which revealed high similarity within each group and distinct differences between groups (**Supplementary Figure S1**). Subsequently, we identified differences in gut microbiota composition among the groups using α-diversity and β-diversity indices. In terms of α-diversity, the IG exhibited the highest phylogenetic diversity, with statistically significant differences compared to the SG (*p*_pd_ = 0.02193, **Figure 1B**). However, there were no significant differences in the Chao, Ace, Shannon, and Simpson indices (*p*_chao_ = 0.1054; *p*_ace_ = 0.07406; *p*_shannon_ = 0.5139; *p*_simpson_ = 0.6561, **Figure 1B**). For β-diversity, significant differences were observed between groups, indicating variations in microbial composition (PCA: R = 2178, *p* = 0.04; PCoA: R= 0.8734, *p* = 0.001, **Figure 1C**). We then assessed the Gut Microbiome Health Index (GMHI) and Microbial Dysbiosis Index (MDI) at the species level of gut microbiome samples to evaluate the health status of the gut microbiota between groups. We found that the GMHI in the 5/6 Nx group was significantly lower than that in the SG (*p*_CG vs. SG_ = 0.01996; *p*_IG vs. SG_ = 0.01996, **Figure 1D**), and the GMHI in the IG was lower than that in the CG (*p*_IG vs. CG_ = 0.03671, **Figure 1D**). The MDI showed that both the IG and CG had increased dysbiosis indices, with the IG exhibiting a more pronounced increase compared to the CG (*p*_CG vs. SG_ = 0.03734; *p*_IG vs. SG_ = 0.01996, **Figure 1E**). This finding suggests that the gut microbiota of mice in the IG was more disrupted than that of mice in the other two groups.

### 3.2 Disrupted gut microbiota aggravates kidney injury in 5/6 Nx mice

**Figure 2:**
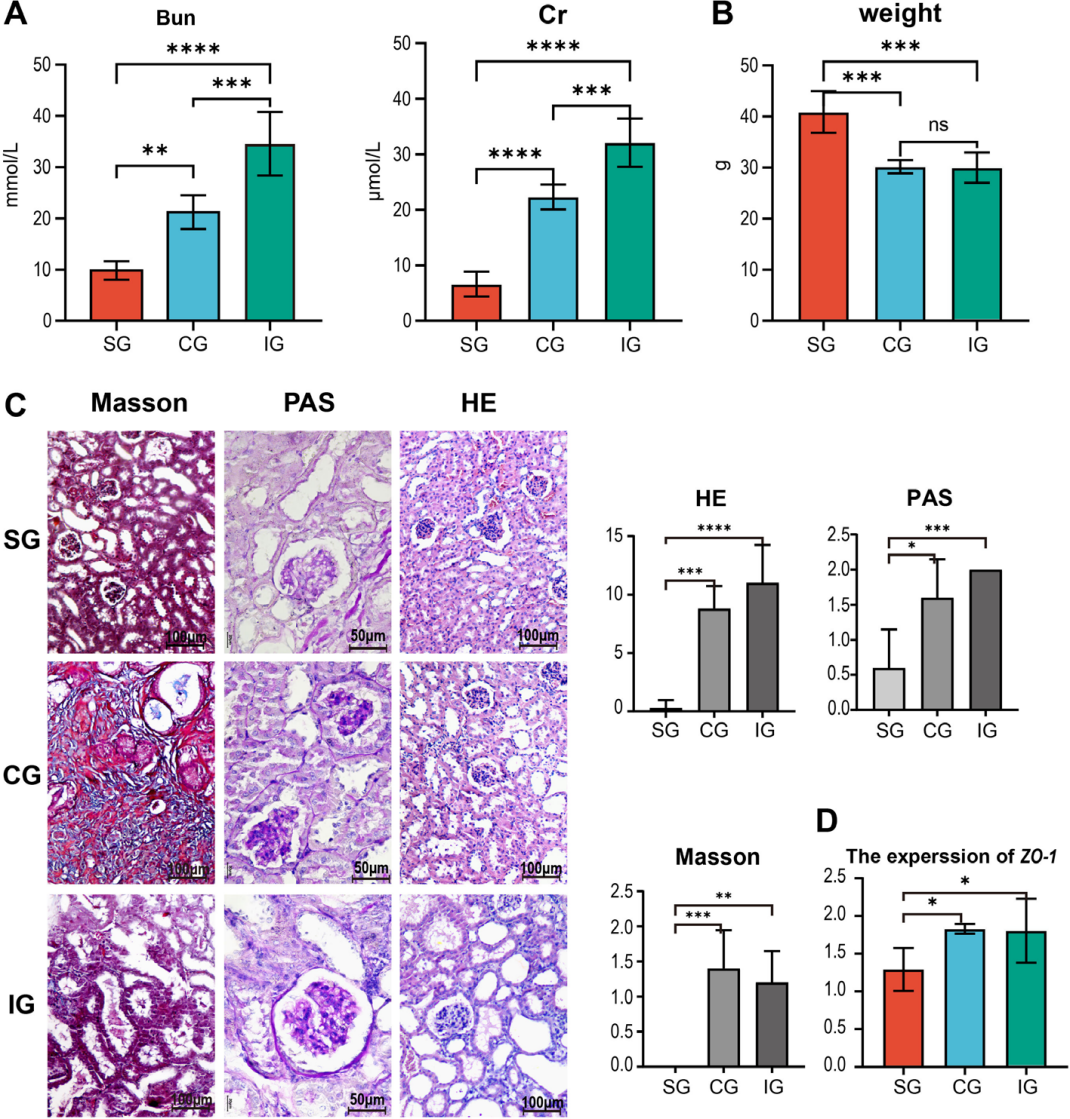
Changes in renal function-related indicators in SG, CG, and IG after Bacillus pumilus intervention. **(A)** Changes in serum creatinine urea nitrogen among the three groups of mice; **(B)** Differences in body weight among the three groups of mice; **(C)** Histological staining of residual kidneys of the three groups of mice by HE, Masson, and PAS, and quantitative statistical analysis of them; **(D)** Relative gene expression of the intestinal tight junction protein *ZO-1* in mice. * indicates *p*<0.05, ** indicates *p*<0.01, *** indicates *p*<0.001, **** indicates *p*<0.0001.

Upon completing the experiment, we analyzed kidney-related indicators and found that serum creatinine and urea nitrogen levels in the IG were significantly higher than those in the SG and CG (Bun: *p*_IG vs. CG_ = 0.0007, *p*_CG vs. SG_ = 0.0027; Cr: *p*_IG vs. CG_ = 0.0008, *p*_CG vs. SG_ < 0.0001, **Figure 2A**). The body weight of the SG mice was higher than that of the 5/6 Nx mice, with no differences observed between the CG and IG (*p*_CG vs. SG_ = 0.0003; *p*_IG vs. SG_ = 0.0003, **Figure 2B**). HE staining revealed that in the CG, the residual kidneys of mice showed an increase in the mesangial matrix within the glomeruli, with mild to moderate dilation and enlargement of the renal tubular lumen in four out of five mice, mild atrophy of the renal tubules in three out of five mice, presence of a small number of hyaline casts, and significant or moderate reduction in the size of renal papillae in three out of five mice compared to the SG. All CG mice exhibited distinct dilation of the renal pelvis, and some mice had mild-to-moderate thickening of the renal capsule, with evident proliferation of fibrous tissue and infiltration of inflammatory cells. In the IG, four out of five mice showed severe dilation and enlargement of the residual renal tubular lumen, severe atrophy of the renal tubules, and mild infiltration of inflammatory cells into the renal interstitium. The size of the renal papillae was significantly or moderately reduced in four out of five mice compared to that in the SG, with the rest being indistinguishable from the CG. To evaluate the differences in the degree of pathological changes among the three groups, we assigned their respective scores. HE staining showed a more pronounced degree of residual renal pathological changes in the IG compared to the CG, with PAS staining revealing a greater degree of mesangial proliferation. Additionally. Masson staining showed more pronounced fibrous proliferation in the capsule (**Figure 2C**); however, these differences were not statistically significant between the CG and IG (*p*_CG vs. SG_ = 0.0002_HE_, 0.0106_PAS_, 0.0004_Masson_; *p*_IG vs. SG_ < 0.0001_HE_, = 0.0009_PAS_, = 0.0015_Masson_, **Figure 2C**). Given that the intestinal mucosal barrier plays a crucial role in the gut-kidney axis, we analyzed the expression levels of the *zonula occludens-1* (*ZO-1*) gene in the intestinal mucosa and observed the status of the mucosal barrier among different groups. We found that *ZO-1* expression was higher in the Nx group than in the SG (*p*_CG vs. SG_ = 0.0361, *p*_IG vs. SG_ = 0.0456, **Figure 2D**).

### 3.3 Correlation analysis of differential gut microbiota and kidney injury in mice from three groups

**Figure 3:**
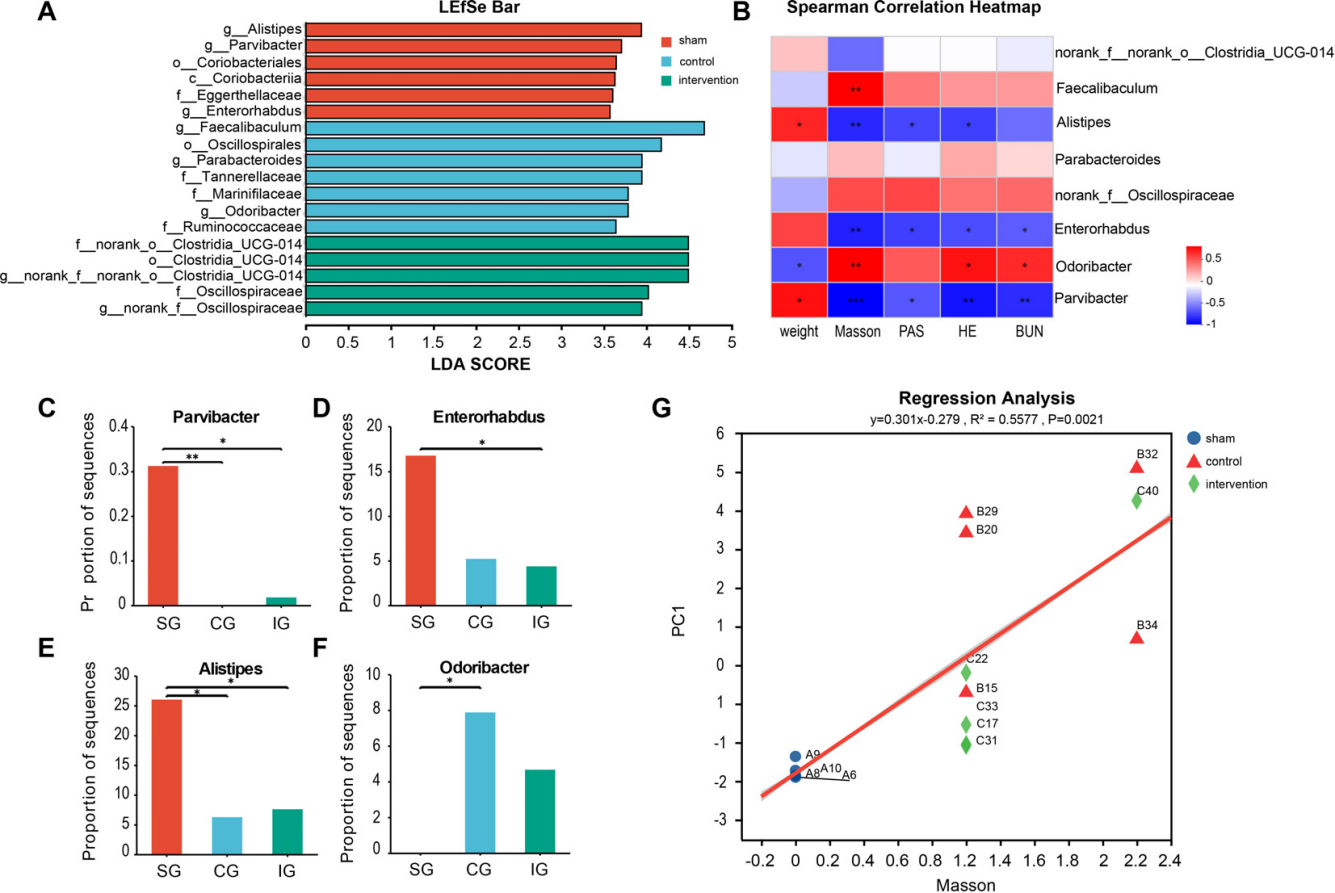
Searching for intestinal microorganisms related to CKD through LEfSe multilevel discriminant analysis. **(A)** Related genus level microorganisms screened by LEfSe differential discriminant analysis; **(B)** Spearman correlation analysis of microbial genus abundance and indicators related to kidney damage; **(C–F)** The relative abundance of significantly different bacterial genera in the intestinal tract of the three groups of mice; **(G)** Linear regression analysis of renal fibrosis and intestinal flora. Positive correlation: red, negative correlation: blue, Spearman correlation analysis was used. * indicates p<0.05, ** indicates p<0.01, *** indicates p<0.001.

To identify distinct gut microbes within the disrupted microbiota, we performed an LDA Effect Size (LEfSe) analysis on the 16s data from the three groups. This analysis identified eight genera-level gut microbes (**Figure 3A**). Specifically, the SG had three genera, including one Bacteroidetes and two Actinobacteria, while the CG had three, comprising one Firmicute and two Bacteroidetes. The IG had two genera, both being Firmicutes. Further, we conducted a Spearman correlation analysis between the differential gut microbes and kidney injury indicators (**Figure 3B**). We selected four differential species for further analysis: *Parvibacter* (*p*_CG vs. SG_ = 0.0047, *p*_IG vs. SG_ = 0.0355, **Figure 3C**), enriched in the SG, *Enterorhabdus* (*p*_IG vs. SG_ = 0.0162, **Figure 3D**), *Alistipes* (*p*_CG vs. SG_ = 0.0226, *p*_IG vs. SG_ = 0.0411, **Figure 3E**), and *Odoribacter* (*p*_CG vs. SG_ = 0.0105, **Figure 3F**), enriched in the 5/6 Nx group. The analysis revealed that *Parvibacter* was negatively correlated with kidney injury, renal fibrosis, glomerulosclerosis, and serum urea nitrogen and positively correlated with body weight. *Enterorhabdus* was negatively correlated with kidney injury, renal fibrosis, glomerulosclerosis, and serum urea nitrogen levels. *Alistipes* was negatively correlated with kidney injury, renal fibrosis, and glomerulosclerosis. *Odoribacter* was positively correlated with kidney injury, renal fibrosis, and serum urea nitrogen and negatively correlated with body weight. These findings suggest that *Parvibacter*, *Enterorhabdus*, and *Alistipes* may be beneficial in CKD, potentially slowing its progression, while an increase in *Odoribacter* may be associated with kidney injury. Additionally, linear regression analysis based on Masson’s staining indicated a high correlation between renal fibrosis and gut microbiota (R^2^ = 0.5577, *p* = 0.0021, **Figure 3G**).

### 3.4 Differences in gut metabolism between 5/6 Nx mice and sham mice

**Figure 4:**
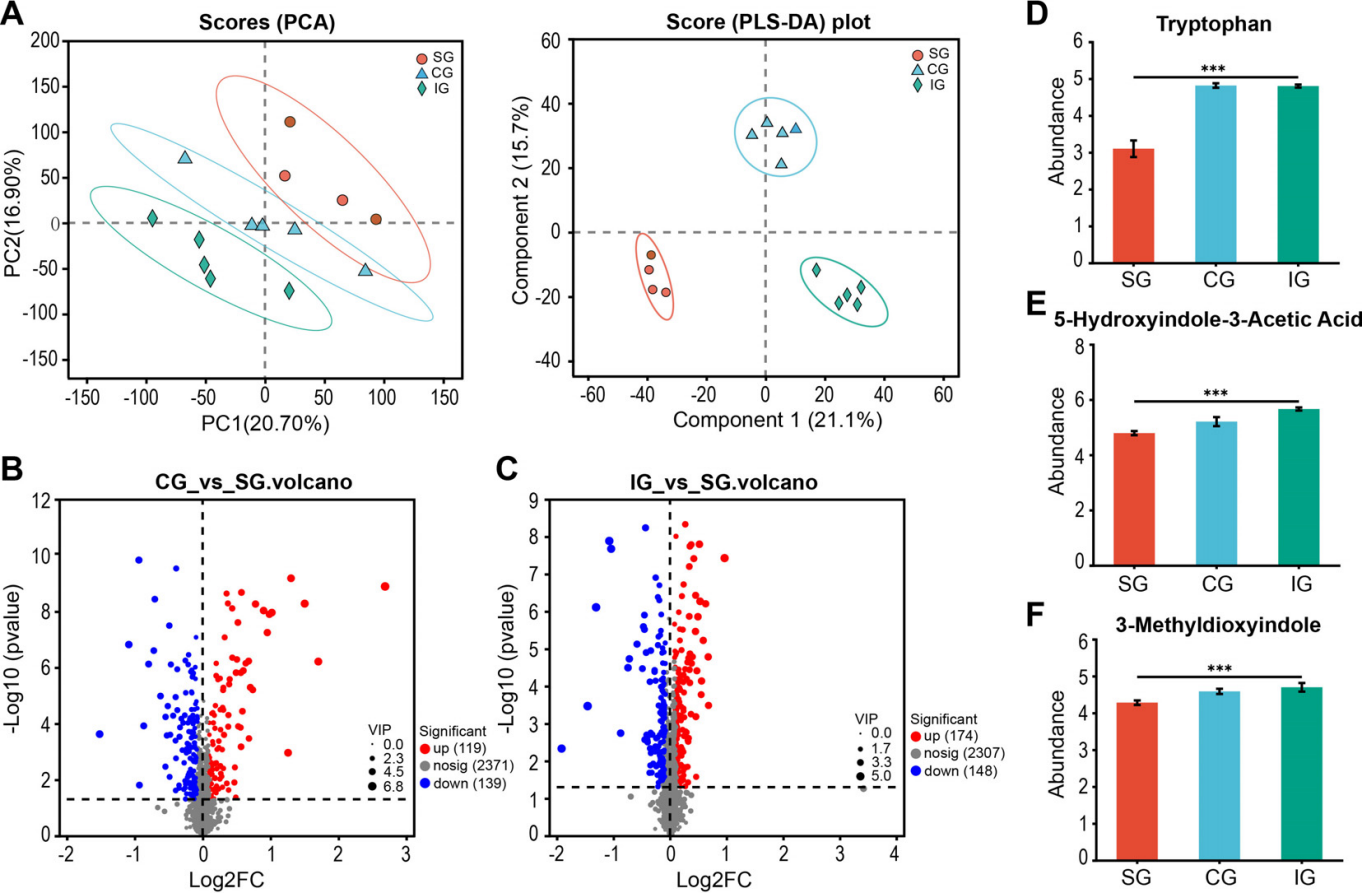
Differences in intestinal metabolites of SG, CG, and IG after Bacillus pumilus intervention. **(A)** PCA and PLS-DA score graph of SG, CG, and IG; **(B)**Volcano graph of differential metabolites in the intestine of mice between CG and SG; **(C)** Volcano graph of differential metabolites in the intestine of mice between IG and SG; **(D–F)** Relative abundance of uremic toxin-related metabolites screened in the intestine. *** indicates p<0.001.

Analysis of the gut metabolomic data from the three groups using PCA and partial least squares discriminant analysis (PLS-DA) revealed significant differences between the groups (**Figure 4A**). The PLS-DA results were Q^2^ = 0.937, R^2^X = 0.649, and R^2^Y = 0.995. The orthogonal PLS-DA (OPLS-DA) demonstrated a significant intergroup dispersion trend, indicating that the variability within groups was smaller than that between groups, with clear intergroup differences (**Supplementary Figures S2A-C**). The parameters for each group suggested that the OPLS-DA model established in this study had good predictive power (**Supplementary Figures S2D–F**). Using criteria of VIP > 1.5 and *p* < 0.05, we identified 258 differential metabolites between the CG and SG (**Figure 4B**) and 322 differential metabolites between the IG and SG (**Figure 4C**). The differential metabolites are listed in **Supplementary Table S1**. These findings suggest that the more disrupted the gut microbiota, the greater the number of differential metabolites compared to the SG. We focused on gut metabolites related to CKD, such as tryptophan (*p*_FDR_ = 0.0024, **Figure 4D**), a precursor of the uremic toxin indole, and its metabolites 5-hydroxyindole-3-acetic acid (*p*_FDR_ < 0.0001, **Figure 4E**) and 3-methyldioxyindole (*p*_FDR_ = 0.0081, **Figure 4F**), which are involved in tryptophan metabolism. These metabolites showed elevated levels in both the CG and IG. Therefore, the disruption of the gut microbiota may have led to an increase in CKD-related uremic toxin metabolites, contributing to CKD progression.

### 3.5 Correlation analysis between disrupted gut microbiota and gut metabolic pathways

**Figure 5:**
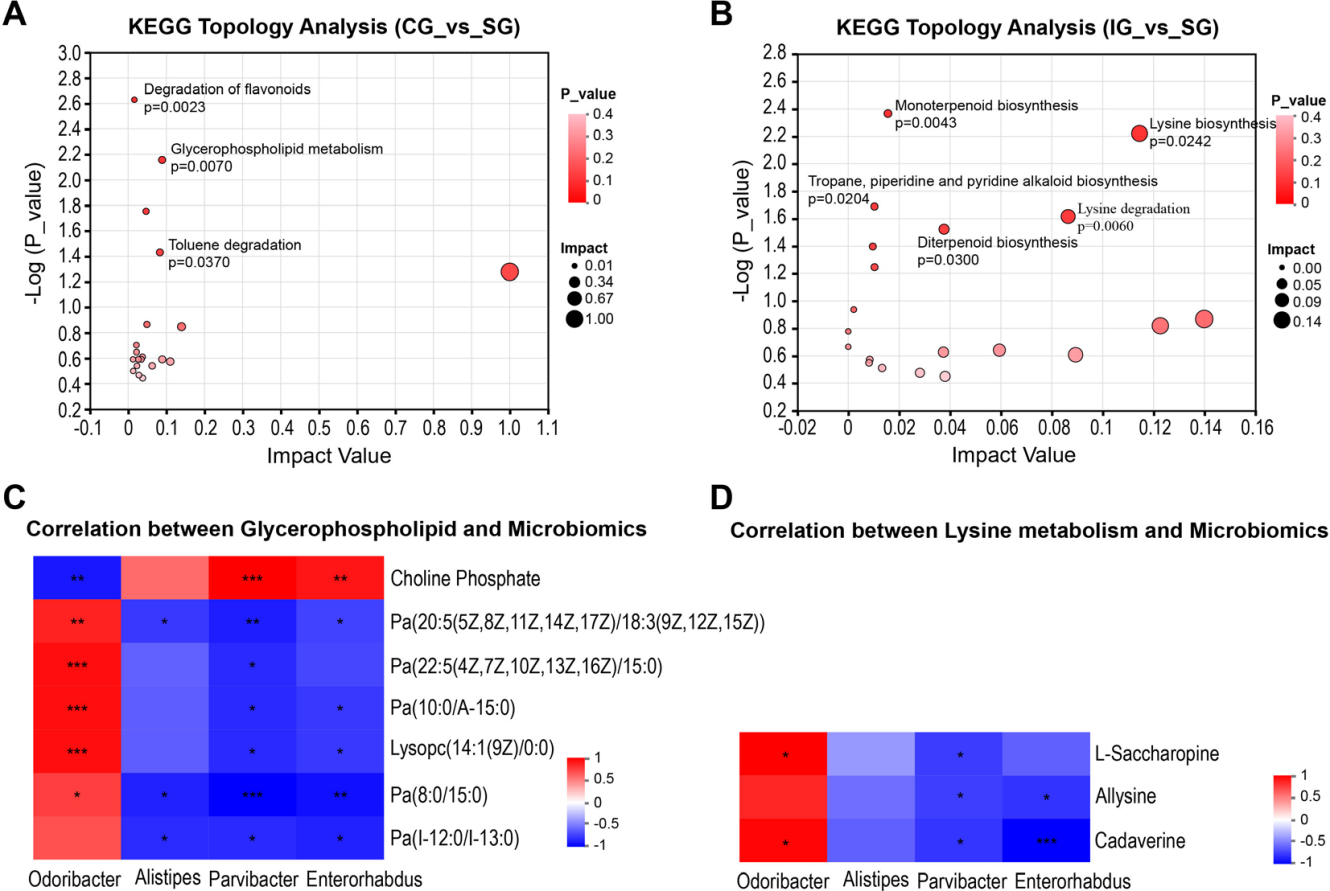
Correlation between metabolic pathways related to kidney injury and intestinal microorganisms. **(A)** Analysis of metabolic pathway differences between CG and SG; **(B)** Analysis of differential pathways between CG and SG; **(C)** Heatmap of the correlation between metabolites related to the glycerophospholipid metabolic pathway and intestinal microorganisms. **(D)** Heatmap of the correlation between metabolites related to the lysine metabolic pathway and intestinal microorganisms. Positive correlation: red, negative correlation: blue, Spearman correlation analysis was used. * indicates p<0.05, ** indicates p<0.01, *** indicates p<0.001.

We conducted a KEGG pathway enrichment analysis on the differential metabolites identified to explore the gut metabolic pathways associated with these metabolites and elucidate potential differences in metabolic pathways between the disease model and healthy groups. The analysis revealed that five metabolic pathways were upregulated in the IG compared to the SG, including the lysine degradation, lysine biosynthesis, diterpenoid biosynthesis, and monoterpenoid biosynthesis pathways, as well as the tropane, piperidine, and pyridine alkaloid biosynthesis pathways. Conversely, the CG showed changes in three metabolic pathways compared to the SG, including the upregulated glycerophospholipid metabolism pathway, downregulated toluene degradation pathway, and flavonoid degradation pathway (**Figures 5A, B**). Integrating KEGG topology with *p*-values and impact values revealed that the upregulated glycerophospholipid metabolism in the CG and the upregulated lysine degradation and biosynthesis pathways in the IG were potentially related to kidney disease progression. We selected the metabolites annotated to these two pathways in KEGG and demonstrated their correlation with differential microbes through association analysis. *Parvibacter* and *Enterorhabdus* were negatively correlated with these two pathways, whereas *Odoribacter* was positively correlated (**Figures 5C, D**). This finding suggests that disrupted gut microbiota may contribute to CKD progression by influencing changes in gut metabolites.

## 4 Discussion

In recent years, the establishment of the gut-kidney axis has drawn attention to the correlation between gut microbiota and kidney diseases. In this study, we transplanted fecal microbes from patients with CKD into mice and found that the gut microbiota could induce clinical disease symptoms in mice similar to those in patients with CKD (Tang et al., 2023). Therefore, we hypothesized that CKD progression likely results from the action of gut microbiota. Selective transplantation of harmful bacteria that are more abundant in CKD patients into a kidney disease mouse model would promote the progression of CKD. Conversely, transplanting probiotics could reduce colonization by harmful bacteria and suppress CKD progression (Wang et al., 2020; Pei et al., 2023). Thus, we deduced that probiotics and harmful bacteria are pivotal to disease progression. Our findings underscore the importance of screening for gut microbes that affect CKD progression as a focus for future studies. *B. pumilus* is not a resident of the human gut microbiota and is widely found in nature; as an exotic species, it is not a traditional pathogenic bacterium of the intestine (Chakravortty et al., 2016). This study employed a gavage of *B. pumilus* to test the following assumptions: first, to investigate whether *B. pumilus*, as an alien microorganism, can alter the gut microbiota status in CKD model mice; second, to determine if the antibiotic-like effects of *B. pumilus* can influence CKD progression in these mice; and third, to identify gut metabolic pathways associated with CKD progression. The aim was to establish a foundation for the clinical application of gut microbiota.

Our study showed that *B. pumilus* intervention disrupted gut microbiota homeostasis in 5/6 Nx mice and exacerbated their kidney burden. This finding contrasts with previous studies showing that *B. pumilus* gavage enhances growth performance and immune indicators in normal mice (Zhang et al., 2021). One possible explanation is that, in the CKD disease state, the resistance of mice decreases (Anders et al., 2013), making them more susceptible to damage from *B. pumilus* (Duc et al., 2004). However, a consistent finding is that *B. pumilus* can increase the α-diversity of the gut microbiota in mice. Although the Shannon, ACE, and Chao indices did not show statistically significant differences in this study, they all exhibited an increasing trend. Additionally, we found that following *B. pumilus* intervention, the levels of metabolites related to the uremic toxin indole in the gut of mice were higher than those in the other two groups, indicating that *B. pumilus* may be associated with an increase in uremic toxins in the gut when kidney damage occurs in mice, which is not observed in normal mice. Therefore, at different stages of disease progression, the regulatory effects of certain gut microbes may have a dual nature of probiotic or harmful bacteria.

By comparing the differences in gut microbiota among mice with different degrees of kidney injury, we identified several groups of gut microbes that may affect CKD progression. *Parvibacter*, a probiotic discovered in the human gut in recent years (Choi et al., 2020), plays an important role in maintaining bile acid homeostasis (Chen et al., 2024; Zhang et al., 2025). It upregulates FabG and baiA, thereby promoting the metabolism of carcinogenic lithocholic acid (Wang et al., 2024a). Additionally, *Parvibacter* regulates lipid metabolism (Li et al., 2022; Wang et al., 2024b) and oxidative stress (Liu et al., 2021), with its main metabolic product being butyrate, a short-chain fatty acid. While limited studies exist on the role of *Parvibacter* in kidney disease, one study revealed that it may prevent peritoneal fibrosis induced by peritoneal dialysis in end-stage kidney disease by enhancing butyrate production, activating PPARγ and inhibiting NF-κB-mediated inflammatory pathways (Wu et al., 2023). In this study, the absence of *Parvibacter* was also associated with an increase in the degree of kidney fibrosis; however, further studies are required to verify this finding. *Enterorhabdus* is a recently identified gut bacterium, and recent studies suggest that it is positively correlated with the health of the colonic mucosal barrier (Mi et al., 2022; Feng et al., 2023). Numerous studies have shown that intestinal mucosal damage is prevalent in the gut-kidney axis in kidney diseases (Guan et al., 2020; Wang et al., 2022). In our study, *ZO-1* expression in SC mice was consistently lower than that in 5/6Nx mice, indicating that in mice with kidney damage, *ZO-1* expression in the gut is active but present in lower quantities. Whether this is due to impaired protein synthesis or increased protein degradation requires further investigation. Additionally, *Enterorhabdus* has been found to be negatively correlated with lipid metabolism (Sun et al., 2020), which aligns with our findings. *Odoribacter* is a harmful gut bacterium identified in CKD and has been proven to significantly increase the urine albumin-to-creatinine ratio in CKD model mice (Jiang et al., 2018), indicating a significant role in kidney damage.

To explore the impact of changes in the gut microbiota on gut metabolism, we analyzed the gut metabolites of the three groups. in order to identify metabolic pathways through which the gut microbiota may affect CKD progression. Metabolomic analysis revealed differences in metabolites among the groups, with an increasing number of differential metabolites as kidney function worsened. Following a KEGG topology analysis of the 5/6 Nx group compared to the SG, we identified three differential metabolic pathways, including two amino acid metabolism pathways and one lipid metabolism pathway, indicating that the gut microbiota may affect CKD progression by influencing lipid and amino acid metabolism in the gut. Glycerophospholipid metabolism is a common metabolic pathway in patients with CKD (Liu et al., 2019; Cai et al., 2021; Yang et al., 2024), but its role in kidney damage has not been reported in the literature. However, metabolites of the glycerophospholipid pathway, such as phosphatidylcholine, phosphatidylethanolamine, phosphatidylinositol, and phosphatidic acid, may maintain cell membrane stability to resist hypoxic stress and reduce cell damage (Xia et al., 2019). In CKD, the glycerophospholipid metabolism pathway may be activated to counteract kidney damage. Lysine is an essential amino acid mainly produced by gut microbiota. Most of the lysine metabolism occurs in the intestinal mucosa. In humans, lysine is rapidly absorbed by the kidney (Tan et al., 2023). The lysine metabolite g-butyrobetaine can be hydroxylated by g-butyrobetaine dioxygenase in the kidney to form carnitine (Vaz and Wanders, 2002). Free carnitine and trimethylamine, the synthetic metabolic product of choline, are precursors of trimethylamine oxide, which is a uremic toxin (Hsu et al., 2020; Zhang et al., 2023). Additionally, the lysine metabolite Nϵ-(carboxymethyl)-lysine induces a decrease in the expression of carnitine palmitoyl transferase 2 in the kidney, leading to fatty acid oxidation damage in mitochondria and kidney fibrosis (Lee et al., 2020). These findings reveal that disrupted gut microbiota can lead to metabolic disorders in the gut, contributing to CKD progression. Our study highlights that continuous loss of gut probiotics and the proliferation of pathogenic bacteria are the main manifestations of gut microbiota disruption, providing potential strategies for the prevention and treatment of CKD in clinical practice.

However, our study has some limitations. First, our sample size was small, which limited our ability to obtain positive information regarding metabolites and metabolic pathways. Second, we obtained only microbial and metabolic information related to kidney disease progression without further validation. Therefore, it is necessary to validate the selected microbes in cellular and animal models to obtain more effective information and to provide a basis for the treatment of patients with CKD in clinical practice. In summary, using the alien microorganism *B. pumilus* to intervene in the gut microbiota, we analyzed the changes in gut microbes and their relationship with CKD. Further, we identified several groups of gut microbes and metabolites that may be related to CKD and elucidated their associations. Our findings offer valuable insights for future studies on the gut-kidney axis.

## Data Availability

The datasets presented in this study can be found in online repositories. The names of the repository/repositories and accession number(s) can be found below: https://www.ncbi.nlm.nih.gov/, SRP546713.
