# Supplementary material for: Disrupted gut microbiota promotes the progression of chronic kidney disease in 5/6 nephrectomy mice by Bacillus pumilus gavage
Source: Front Cell Infect Microbiol. 2025 Mar 18;15:1548767. doi: 10.3389/fcimb.2025.1548767 (PMC11959065; doi:10.3389/fcimb.2025.1548767)
Supplement: Supplementary file 1 [file DataSheet1.docx]

Supplementary Material

# Supplementary Figures


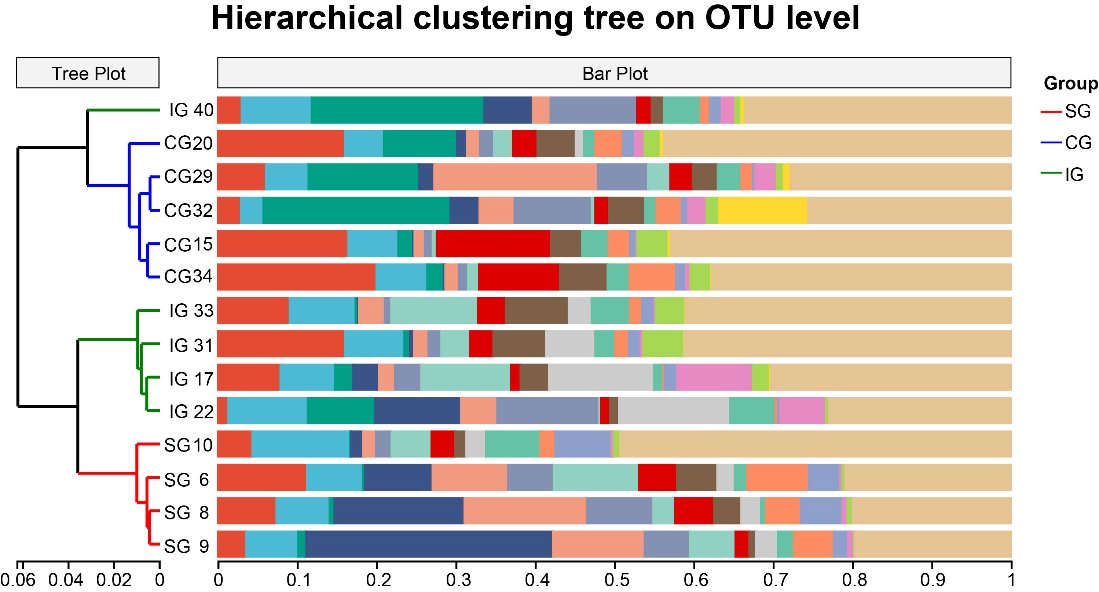


**Supplementary Figure 1.** Hierarchical clustering analysis chart of the SG, CG and IG. The numbers behind group names are ear tag figures.


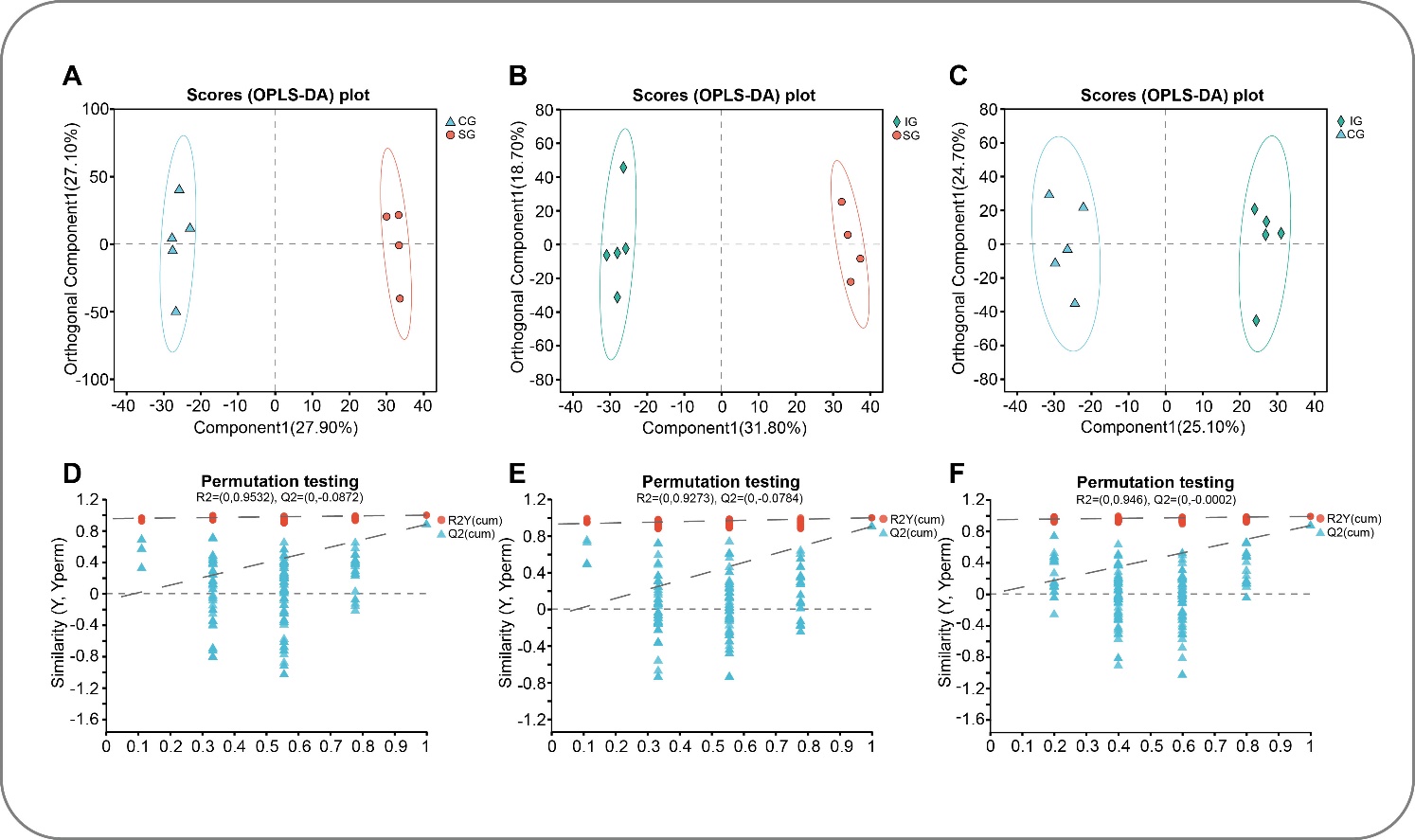


**Supplementary Figure 2.** (A-C) OPLS-DA score plots for each group within SG, CG and IG; (D-F) Model validation plots for the SG, CG, IG.
